# Characterizing Experiences With Hikikomori Syndrome on Twitter Among Japanese-Language Users: Qualitative Infodemiology Content Analysis

**DOI:** 10.2196/65610

**Published:** 2025-02-24

**Authors:** Misa Ashley Uchiyama, Hirofumi Bekki, Tiana McMann, Zhuoran Li, Tim Mackey

**Affiliations:** 1 Global Health Policy and Data Institute San Diego, CA United States; 2 Kyushu Medical Center Fukuoka Japan; 3 Global Health Program Department of Anthropology University of California San Diego La Jolla, CA United States; 4 S-3 Research San Diego, CA United States

**Keywords:** hikikomori, social withdrawal, hikikomori syndrome, mental health, social isolation

## Abstract

**Background:**

*Hikikomori* syndrome is a form of severe social withdrawal prevalent in Japan but is also a worldwide psychiatric issue. Twitter (subsequently rebranded X) offers valuable insights into personal experiences with mental health conditions, particularly among isolated individuals or hard-to-reach populations.

**Objective:**

This study aimed to examine trends in firsthand and secondhand experiences reported on Twitter between 2021 and 2023 in the Japanese language.

**Methods:**

Tweets were collected using the Twitter academic research application programming interface filtered for the following keywords: “#引きこもり,” “#ひきこもり,” “#hikikomori,” “#ニート,” “#脱ひきこもり,” “#不登校,” and “#自宅警備員.” The Bidirectional Encoder Representations From Transformers language model was used to analyze all Japanese-language posts collected. Themes and subthemes were then inductively coded for in-depth exploration of topic clusters relevant to first- and secondhand experiences with *hikikomori* syndrome.

**Results:**

We collected 2,018,822 tweets, which were narrowed down to 379,265 (18.79%) tweets in Japanese from January 2021 to January 2023. After examining the topic clusters output by the Bidirectional Encoder Representations From Transformers model, 4 topics were determined to be relevant to the study aims. A total of 400 of the most highly interacted with tweets from these topic clusters were manually annotated for inclusion and exclusion, of which 148 (37%) tweets from 89 unique users were identified as relevant to *hikikomori* experiences. Of these 148 relevant tweets, 71 (48%) were identified as firsthand accounts, and 77 (52%) were identified as secondhand accounts. Within firsthand reports, the themes identified included seeking social support, personal anecdotes, debunking misconceptions, and emotional ranting. Within secondhand reports, themes included seeking social support, personal anecdotes, seeking and giving advice, and advocacy against the negative stigma of *hikikomori*.

**Conclusions:**

This study provides new insights into experiences reported by web-based users regarding *hikikomori* syndrome specific to Japanese-speaking populations. Although not yet found in diagnostic manuals classifying mental disorders, the rise of web-based lifestyles as a consequence of the COVID-19 pandemic has increased the importance of discussions regarding *hikikomori* syndrome in web-based spaces. The results indicate that social media platforms may represent a web-based space for those experiencing *hikikomori* syndrome to engage in social interaction, advocacy against stigmatization, and participation in a community that can be maintained through a web-based barrier and minimized sense of social anxiety.

## Introduction

### Background

*Hikikomori* syndrome, a form of severe social withdrawal largely characterized as experienced among adolescents and young adults in Japan, has recently gained increased attention as a global mental health concern [1]. Importantly, variability in reported *hikikomori* prevalence in countries and regions such as China, Hong Kong, South Korea, Singapore, Nigeria, the United States, and Taiwan may reflect different cultural distinctions of *hikikomori* inclusion criteria, study-specific assessments, and study-specific enrollment methods [2,3]. Nevertheless, increasing prevalence continues to challenge the notion that *hikikomori* is specific to the Japanese context and provides emerging evidence that this phenomenon is widespread cross-nationally [2,4]. Importantly, this form of extreme and persistent social isolation and withdrawal can be viewed as a complex sociocultural mental health phenomenon influenced by a variety of factors, such as economic and employment conditions, social norms and expectations, technology access and use, and changing attitudes toward acceptable social interaction (such as changes in interpersonal dynamics caused by the social isolation experienced during the COVID-19 pandemic) [5,6].

*Hikikomori* (derived from the verb *hik* [引き], which means to withdraw, and *komori* [籠り], which means to be inside) was originally coined by Japanese psychologist Tamaki Saito in 1998. The term was originally operationalized to refer to an individual who has stopped going to school (*futoukou* [不登校]) or work (*neeto* [ニート]) and has remained at home for a duration of >6 months [7]. A consensus on a standardized definition of *hikikomori* has not been reached, contributing to challenges in measuring the phenomenon, but a commonly used set of criteria was created in 2003 by the Japanese Ministry of Health, Labor, and Welfare (JMHLW) [1,4]. The JMHLW criteria have since been updated with the most recent 2010 definition, which describes *hikikomori* as a result of various factors, such as avoiding social participation (such as schooling, including compulsory education; employment, including part-time jobs; and other interactions outside the home), which in principle has continued under the condition of being housebound for a period of >6 months (this may include leaving the home while still avoiding interactions with others [8]). A later definition in 2020 by Kato et al [9] proposed updated diagnostic criteria for *hikikomori* as a pathological social withdrawal or social isolation in which the essential feature is physical isolation in one’s home and for which the person needs to meet the criteria of (1) marked social isolation in one’s home, (2) duration of continuous social isolation of at least 6 months, and (3) significant functional impairment or distress associated with the social isolation. Furthermore, many studies have found that patients with *hikikomori* syndrome often had experiences with bullying, peer rejection, or dysfunctional family life and were prone to internet addiction [10,11].

However, until recently, *hikikomori* was understood as a culture-bound phenomenon unique to Japan, reported to affect an estimated 1.2% of the population and over a quarter of students based on household survey data [2,12,13]. Although the causes and risk factors for *hikikomori* are not completely known, many studies have highlighted aspects of Japanese society and culture that enable *hikikomori* features and may account for the especially high number of *hikikomori* cases reported in Japan. Sociocultural factors such as *amae* (甘え), the Japanese term for codependency in parent-child relationships; the tendency of overprotection and indulgence of children by parents; the high-pressure environment created by the Japanese educational system; the need to conform to others and norms; and the challenging job search process for young adults often leading to identity distress have all been hypothesized to be causes of or risk factors for *hikikomori* [14-16]. Furthermore, the idea of isolation has been prominent in Japanese society for centuries. It has been seen as a way of life commonly represented in history with tales of mysterious mountain recluses and hermits [17]. However, numerous *hikikomori*-like situations and the lack of standardized diagnostic methods have made identifying *hikikomori* challenging in Japan [1].

Previous studies have attempted to carry out clinical interviews with families or study individuals who have sought help from public health centers for *hikikomori* syndrome, but the underlining challenges of social reclusion have also made *hikikomori* research and recruitment difficult [12]. Those who experience social and geographic isolation often feel unable to discuss mental illness openly due to the fears of stigma and may feel more comfortable sharing their experiences on the web [18,19]. In response, researchers have leveraged social media platforms as a source of self-reported health information that can be analyzed for stigmatizing issues and topics discussed among hard-to-reach populations, including generating insights specific to certain demographics and geographies [20,21]. Despite this possible application to *hikikomori* research, existing studies using web-based sources of data are limited and have primarily focused on exploring *hikikomori* through Western tweets outside of Japan and tweets in Japanese with limited keywords or have studied *hikikomori* alongside other mental health phenomena [22-24].

### Objectives

Importantly, *hikikomori* has evolved since its introduction and original classification in 2003. While initially classified as a cultural syndrome in the 2019 version of the *Diagnostic and Statistical Manual of Mental Disorders*, it has since been included in the appendix of the 2022 *Diagnostic and Statistical Manual of Mental Disorders*, indicating that it will become a formal addition to the volume [25]. These changes may be a result of increasing public awareness, increased willingness to discuss mental health topics, and destigmatization yet can cause an expansion or inflation of the clinical meaning of *hikikomori* [26]. In response, this exploratory study sought to expand knowledge on *hikikomori* syndrome with a focus on Japanese-language social media posts from Twitter (now known as X), a platform that is popular among Japanese web-based users. Furthermore, no study, to the best of our knowledge, has examined *hikikomori*-related data after 2020 (the start of the COVID-19 pandemic) despite the pandemic contributing to a rise in social isolation due to public health measures mediated by increased use of social media for social interactions [27,28]. We also sought to source more diverse data and web-based discussions by including additional keywords in Japanese, such as more casual terms, closely related words, and synonyms related to *hikikomori*. Finally, this study focused on firsthand and secondhand experiences self-reported by Twitter users and how those experiencing or who have had experience with *hikikomori* interact on the platform. The results of this study can provide insights into how the Japanese *hikikomori* population and their caregivers use social media to discuss this condition and promote a better understanding of primary concerns and behaviors that can help destigmatize this growing condition.

## Methods

### Data Collection

We first conducted manual searches of *hikikomori* posts on Twitter to identify keywords and hashtags associated with *hikikomori* conversations and mentions in the Japanese language. From this initial search, we identified a set of *hikikomori* keywords that Japanese-language Twitter users commonly used in web-based discussions regarding *hikikomori* syndrome (Multimedia Appendix 1). This initial set of keywords included nonspecific *hikikomori* keywords such as stopping going to school (*futoukou* [“不登校”]) or work (*neeto* [“ニート”]) and staying at home (“自宅警備員”), all terms that are considered a similar social phenomenon to *hikikomori* and often exhibit similar characteristics of social isolation as those included in the definition of *hikikomori* by the JMHLW. This approach was also supplemented by conducting an analysis of Google Trends data for related topics and queries associated with the Japanese-language spelling of *hikikomori* (“引きこもり”) from 2004 to the present, which identified additional related topic keywords used in this study.

After the study keywords were finalized, the Twitter application programming interface (API) was used to collect all Twitter posts (ie, tweets) in 50 languages, including Japanese and English. We then limited the data to the Japanese language only (ie, filtered data in the JSON language field *LANG* for the *JA* [Japanese] attribute); removed all retweets; and only included data over a 2-year time frame from January 1, 2021, to January 1, 2023. For the academic API data collection, the API had a limit of 300 queries per a 15-minute window, so the *next_token* feature was used to collect data continually between all queries to ensure that all available data in the given period were collected based on the API settings. The Twitter data field categories analyzed for this study primarily consisted of text, including the following fields: text, link, ID associated with the tweet, username (deidentified and not disclosed in this study), user link, author ID, API type, geolocation (latitude and longitude, if available), and tweet creation time and date. Data collection took place in June 2023.

### Topic Modeling

Before topic modeling, any retweeted tweets were removed, and only unique tweets with a unique Twitter ID were analyzed. Due to the relatively large volume of data collected, we applied a natural language processing approach to group tweets into relevant thematic clusters. For this corpus of tweets, we used BERTopic, which is a topic modeling technique that leverages Bidirectional Encoder Representations From Transformers (BERT), and class-based term frequency–inverse document frequency, a statistical measure of the importance of words to a particular group of text, to create dense clusters allowing for easily interpretable topics while keeping important words in the topic descriptions. BERTopic then produced an output of data using the k-means algorithm, which includes the sum of the posts into a predetermined number of *k* clusters (*k*=10) based on the posts’ semantic similarities and groups text containing the same word-related themes into the same clusters. BERTopic was selected due to its use in previous work that has analyzed large-scale Twitter data, its general utility in analyzing unexplored themes that lack existing training data, and utility for the overall exploratory nature of this study’s aims [29,30]. Importantly, when compared against other traditional topic models, BERTopic has resulted in a better performance on both topic coherence and topic diversity on Twitter data [31]. Hence, BERTopic methodically has better utility to group tweets that are specifically relevant to *hikikomori* while reducing the potential for noise in selected clusters by providing more accurate and contextually relevant tweet conversational groupings. This study used BERTopic version 0.6.0 with Python version 3.7 (Python Software Foundation).

**Figure 1:**
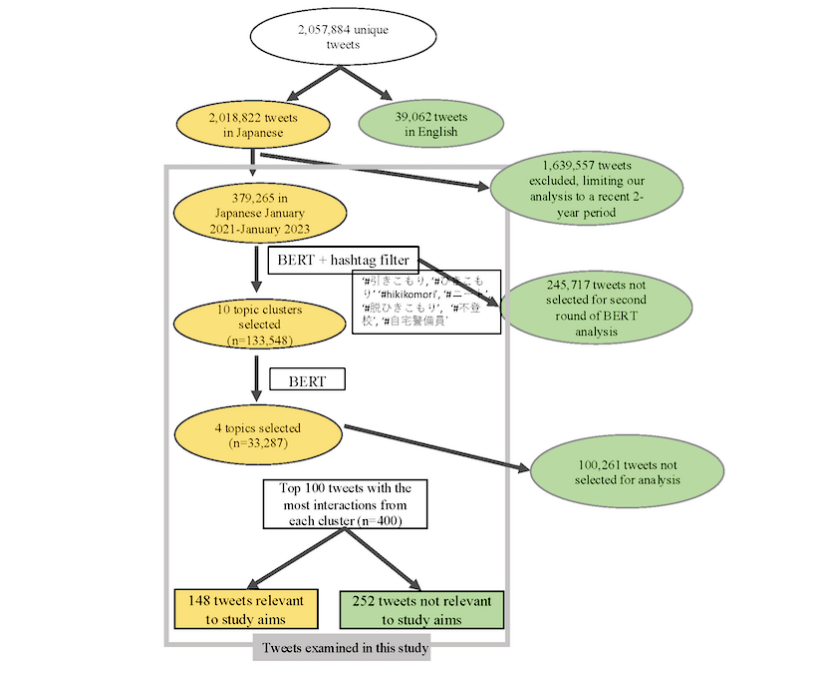
Inclusion criteria and study methodology. BERT: Bidirectional Encoder Representations From Transformers.

For the purposes of analyzing tweets specific to the aims of this study, BERTopic was executed in 2 phases: an initial round on the full dataset after data cleaning followed by a second round of focused analysis on relevant, selected topics. Data cleaning performed before the BERTopic analysis included removing punctuation and stop words in posts for optimized BERTopic grouping output. For the initial BERTopic analysis, we ran both 1- and 2-gram analyses on the same dataset to obtain the most visibility of content in our dataset. From both results, we selected 10 clusters in total for review. From those 10 clusters, we limited the data to the following hashtags—“#引きこもり” (“*hikikomori*”), “#hikikomori,” “#ニート” (“*neeto*”), “#脱ひきこもり” (“stopping *hikikomori* lifestyle”), “#不登校” (“prolonged absence from school”), and “#自宅警備員” (“home security guard”)—to further reduce the data size (see Figure 1 for a summary of the study methodology). With results from these data, a second round of BERTopic analysis was run on the initial 10 topics using a 2-gram BERTopic analysis, and the output topic clusters were reviewed for a final set of 4 clusters that were selected due to their high relevance to *hikikomori* topics. Before manual annotation, we reverted the cleaned posts to their original text with punctuation and stop words to ensure complete comprehension of each post as initially posted. The top 100 tweets with the most interactions from users within the 4 relevant clusters were then selected for manual annotation.

### Qualitative Content Analysis

The objective of this study was to conduct an in-depth analysis of themes associated with self-reported firsthand and secondhand experiences with *hikikomori* as expressed by Japanese-language Twitter users. For the purpose of study analysis, we relied solely on self-reported *hikikomori* experiences perceived by users and those who observed or interacted with individuals who perceived that they were experiencing *hikikomori* rather than reported verified clinical diagnoses. Hence, there may be variation in the clinical definition of *hikikomori* and self-reported *hikikomori* experiences detected in this study. Our content analysis focused on detecting themes related to firsthand or secondhand knowledge, attitudes, and experiences related to *hikikomori* syndrome or associated characteristics of severe social withdrawal. To classify the content of the collected tweets following topic modeling and topic cluster selection, 2 coders who were native Japanese speakers (the first and second authors, MAU and HB) first independently used a binary coding approach to identify tweets that were relevant to the study aims and excluded tweets that did not fall under the criteria of *hikikomori* syndrome knowledge, attitudes, or experiences self-reported by Twitter users (eg, discussions related to other health or psychological conditions, news articles, statistics or opinions about *hikikomori*, and other topics that used the *hikikomori* term but were not related to the syndrome, hereinafter referred to as *noise*). The primary focus of this study was to identify tweets that met the following conditions: (1) were posted by a Twitter account that appeared to be an individual account (eg, not an organizational, news, or botlike account); (2) self-reported firsthand knowledge of, attitudes toward, or experiences with *hikikomori* syndrome; or (3) self-reported secondhand knowledge of a friend, family member, caregiver, or other social contact that experienced *hikikomori* syndrome.

Discussion of *hikikomori* syndrome or related topics (such as “不登校” or “ニート,” which translate to prolonged absence from school or work in English) in the post content, along with pronouns (such as “僕,” “私,” and “俺,” which translate to *I* or first-person pronouns in English, or “彼,” “彼女,” and “あの子/子供,” which translate to *him* or *her*, *that* or *my child*, or second-person pronouns in English) or other form of reference to the user themselves, signaled relevance as a firsthand or secondhand account. After applying this binary coding scheme for inclusion and exclusion, we then used a general inductive coding approach to conduct in-depth qualitative coding of all relevant tweets selected (hereinafter referred to as *signal tweets*). First, a sample of signal tweets were inductively coded by MAU and HB, and notes were taken on the general themes of posts, from which an initial code list was created focusing on specific *hikikomori* experiences, behaviors, and societal factors associated with *hikikomori* syndrome. Next, formal coding of all signal tweets was conducted using refined codes and developed subcodes. Finally, MAU and HB reviewed the final coded dataset, and the authors reconciled differences in code definitions and application with senior author TKM, also a native Japanese speaker. MAU and HB coded all posts independently and achieved high intercoder reliability for Twitter thematic classification (Cohen κ=0.95).

On the basis of the content of the collected tweets, all detected themes were classified into three major themes: (1) clinical symptoms, with anxiety (“不安障害” and “パニック障害”), social isolation (“社会的孤立” and “ぼっち”), depression (“うつ病”), self-harm (“死にたい,” “リスカ,” and “自殺”), and developmental and learning disorders (“発達障害” and “学習障害”) as subthemes; (2) social determinants, with school (“不登校”) and work (“ニート”) as subthemes; and (3) awareness, with 1 subtheme detected, education. Descriptive statistics of data characteristics and distribution of the volume of topics coded were also carried out.

### Topic Interaction Analysis

To further analyze the levels of user interactivity with different topics related to *hikikomori* experiences self-reported by Japanese-speaking Twitter users, we also examined the volume of users’ interaction behavior for all signal tweets. The interactivity with tweets was determined using the number of likes, retweets, comments, and favorites for the tweets analyzed.

### Ethical Considerations

This study was exempt from institutional review board approval in accordance with the Common Rule as all data were publicly available and any user-generated data did not include individually identifiable information, and the results are paraphrased and deidentified.

## Results

### Overview

A total of 2,057,884 tweets were collected from Twitter from February 13, 2009, to June 23, 2023 (n=2,018,822, 98.1% tweets in Japanese and n=39,062, 1.9% tweets in English), based on the method of data collection used. After the exclusion of English-language tweets and limiting our analysis to a recent 2-year period (to examine the more recent discourse concerning *hikikomori* and discussions centered on the general time frame of the COVID-19 pandemic), 18.43% (379,265/2,057,884) of the tweets in Japanese from January 2021 to January 2023 were included for full analysis. Our results are organized into a description of the output topics selected and the qualitative content analysis of specific tweets in each selected cluster.

### Topic Selection and Features

The initial 10 topics selected after the first round of BERT analysis all had overlapping themes of mental health and withdrawal from society and high frequency of *hikikomori*-related terms. Frequently mentioned terms included a variation of the term *hikikomori* (“ひきこもり” or “ 引きこもり”); words associated with mental health conditions such as depression (“うつ病”); and other related terms associated with being socially isolated, such as not being able to go to school (“不登校”) or work (“ニート”), which provided a preliminary indication that the cluster included conversational groupings related to *hikikomori* behavior and user perceptions. Other terms identified included *hikikomori*-related services or lifestyle, such as unconventional schooling methods (“free school” [“フリースクール”]), or topics such as gaming, gaming livestreams (“ゲーム 実況”), or web-based platforms (eg, “YouTube”) that were suspected as also indicating a *hikikomori* lifestyle. In addition, the presence of more colloquial or casual terms was thought to be more associated with firsthand and secondhand accounts. Following the second round of BERTopic analysis, all *hikikomori*-related themes appeared to be categorized into 1 of the 4 topic clusters selected for the final analysis (topic 1, topic 7, topic 11, and topic 14).

### Final Topics for Manual Annotation

The first topic selected as output using BERTopic (topic 1) was selected due the frequency of words such as “お悩み相談” (“consulting for advice”), “話したい” (“want to talk”), and “不登校さんとがりたい” (“seeking connection with someone unable to go to school”), which indicate that tweets in the cluster had a focus on seeking help or connections within the community of those with similar experiences. Phrases such as “必ず返信します” (“I will definitely reply”) suggest the practical uses of Twitter as a platform to encourage and facilitate user interaction. Words such as “フリースクール” (schools dedicated to children who fail to fit into conventional school systems in Japan) and “カウンセリング” (“counseling” or “therapy”) allude to discussion of the services that are available for those experiencing *hikikomori*, including “精神疾患” (“psychological disorder”) and “発達障害” (“developmental disorder”), which further suggests that there is discussion of related disorders and *hikikomori*’s associated impacts. Although there were many possible subtopics in the cluster, there was an overall emphasis on seeking help and exchange of information about the syndrome.

Our second topic selected (topic 7) was focused on mental health, containing topics such as depression (“うつ病”) and suicide (“'死にたい”), as well as other words or ideas that are closely related, such as bullying (“いじめ”) or feelings of uncertainty or mental instability (“不安”). Words in the cluster were collectively pessimistic or had negative connotations. Phrases such as “どうでもいい報告をする” (“will report something of no use”) may indicate that Twitter users in this cluster of tweets feel as if their words have little impact or may be meaningless, which is in concordance with the overall topic of depression and mental health. This cluster contained tweets related to escaping reality and searching for a place to cope, which indicates that platforms such as Twitter may serve as a conversation space for those who are experiencing *hikikomori* syndrome and other related mental health disorders.

The third topic selected (topic 11) centered on secondhand accounts of *hikikomori* experience, most of which came from parents or guardians of youths or minors experiencing “不登校の親” (“parents of child unable to go to school or hikikomori”). Words in the cluster such as “いじめ” (“bullying”) and “子育て” (“raising children”) indicate caregivers’ concerns about their children regarding their experiences. This cluster is also characterized by a large portion of keywords related to an unwillingness of youth experiencing *hikikomori* to go to school (“学校行きたくない”), which aligns with the social isolation factor that characterizes *hikikomori* syndrome, suggesting the very closely interlinked ideas of *hikikomori* syndrome and “不登校” (“inability to go to school”). More general terms such as “中学生” (“middle schooler”) or “小学生” (“elementary schooler”) that were included in the cluster suggest that education and school are main topics of discussion and indicate at what grade levels children may be first experiencing *hikikomori* syndrome.

The final topic selected (topic 14) was similar to topic 11 in that it also had a focus on caregivers of youth experiencing *hikikomori*. Many similar words, such as “不登校の親” (“parent of a child unable to go to school”) and “子育て” (“raising children”), were also included in this cluster. However, topic 14 had a more specific focus on solutions to struggles, including alternatives to public school—indicated by words such as “フリースクール” (schools dedicated to children who fail to fit into conventional school systems in Japan) and “家庭教師” (“home or private tutoring”). In addition, words such as “メンタルヘルス” (“mental health”) suggested more discussion and awareness associated with *hikikomori* syndrome. Overall, this cluster highlighted the caregivers’ crucial role as the connection between those with *hikikomori* syndrome and the outside world through platforms such as Twitter, discussion and advocacy, and seeking of opportunities for support and services.

### Content Analysis

After the initial round of BERTopic analysis, 10 topic clusters (n=133,548 tweets) were selected as relevant to the study aims and underwent an additional round of BERTopic analysis. Following the second round of running BERTopic, 4 topic clusters (n=33,287 tweets from 6403 unique users) were determined to be relevant to the study aims. From these, the top 100 tweets with the most engagement (measured using the sum of the likes, comments, and retweets) from each of the relevant 4 topics (n=400) were extracted and manually annotated for inclusion or exclusion, of which 37% (148/400) of tweets from 89 unique users were identified as relevant to *hikikomori* experiences. Of these 148 relevant tweets, 71 (48%) were identified as firsthand accounts (eg, individuals who currently had or had recently had direct experience with *hikikomori* syndrome), and 77 (52%) were identified as secondhand accounts (eg, parents or guardians of individuals with *hikikomori* syndrome who often lived in the same household). Our qualitative analysis and inductive coding approach derived 8 topics based on our 3 parent categories. All the detected topics were first classified into the 3 parent domains: clinical symptoms (58/148, 39.2%), social determinants (111/148, 75%), and awareness (33/148, 22.3%; see Table 1 for a description and example tweets of the themes and subthemes).

Posts identified within the clinical symptoms domain were characterized by discussions related to explicit or implicit descriptions or mentions of mental health conditions or symptoms related to *hikikomori* syndrome, including both firsthand accounts (52/58, 90%) and secondhand accounts (6/58, 10%). Within this parent domain, frequently discussed symptoms included anxiety (2/58, 3%), social isolation (32/58, 55%), and depression (24/58, 41%). These subthemes represent symptomology most commonly associated with *hikikomori* syndrome. However, additional subthemes within this domain included self-harm (7/58, 12%) and developmental and learning disorders (3/58, 5%) mentioned and discussed alongside *hikikomori* syndrome, suggesting that these other disorders and symptoms may be additional or emerging symptoms associated with *hikikomori*. Frequent examples of posts within the social isolation subtheme included a stated desire for community support, friends, or others with similar experiences on the platform alongside mentions of their physical and social isolation (eg, isolating within their home). For example, users sought friendships with like-minded individuals, often asking to connect with those within a specific age range (eg, “中学生” [“middle schooler”]) or someone with a particular experience (eg, “不登校” [“someone unable to go to school”]). Within the subtheme of depression, individuals often used the platform to rant or openly vent about their depressive symptoms and as a place of expression. Tweets within this subtheme were more of a snapshot of an individual’s emotions rather than a recollection of an event or informational content. These tweets often included the hashtag “#うつ病” (“depression”). Self-harm had overlap with our depression subtheme but diverged in its explicit mentions of self-harm through suicide or wrist cutting. Tweets frequently included the hashtag “#死にたい” (“want to die”). Our final subtheme, developmental and learning disorders, was frequently mentioned specifically by caregivers. Disorders were cited as factors that led to the *hikikomori* lifestyle or the inability to go to school as conditions accompanying *hikikomori* syndrome or as hindrances to daily life. Other disorders that are diagnostically unrelated were also mentioned alongside *hikikomori* syndrome. Tweets within this subtheme frequently included the hashtags “＃発達障害” (“developmental disorder”) and “#学習障害” (“learning disability”).

**Table 1:** Explanation and paraphrased examples of the identified hikikomori parent domains and topic subcodes detected on Twitter generated from content analysis (N=148).

Parent domain and subtheme		Subtheme description	Example tweet (original+translation)	Tweets, n (%)	
**Clinical symptoms**					58 (39.2)
	Anxiety	Content that describes anxious tendencies or uses terms that relate to them (ie, 不安 or “anxious”)	不安が増す。(“Anxious thoughts are increasing.”) 何に追われてるのかわからないが、 お金、将来、健康 (“I don’t know what is stressing me out, but money, future, health”) 家にじっとしているともういてもたってもいられなくなる。。 (“If I stay at home, I won’t be able to sit still.”) 読書やテレビを観ても頭に入らない。(“I can’t focus when I read or watch TV.”) 参ってます。(“I’m exhausted.”) #鬱 #うつ病 #適応障害 #パニック障害 #不安障害 #不安 #人間関係 #心療内科 #孤独 #ひきこもり #休職 #会社関係 (“#Depressed #Depression #Adjustment disorder #Panic disorder #Anxiety disorder #Anxiety #Human relations #Psychotherapy #Loneliness #Hikikomori #Leave of employment #Co-worker relations”)	2 (1.4)	
	Social isolation	Content that describes social isolation as part of the user’s lifestyle	休職して孤独な年末年始を目前に(“Taking a leave of absence and facing the lonely New Year holidays”) 家のものを断捨離した (“I got rid of things at home”) 少しスッキリした社宅の中 (“Inside the slightly empty company housing”) 少し気分が晴れた (“I feel a little better”)	32 (21.6)	
	Depression	Content that displays depressive thoughts or episodes that reflect a nonprogressive and pessimistic mindset of the user	買いたいもの、欲しいものなんて買えないし、バイトする気力は無いし、そもそも生きたい理由もないから、何もできない。(“I can’t buy anything I want, I don’t have the energy to work part-time, and I have no reason to want to live in the first place, so I can’t do anything.”) 生きているだけで惨めな思いをする。(“Just being alive makes me feel miserable.”) 頑張れないし、苦しいし、生きていても迷惑かけるだけだから死にたいって思うのは「甘え」なのだろうか。(“Am I acting like a spoiled child to think that I want to die because I can’t do my best, it’s painful, and even if I live I’ll only cause trouble?”) #ニート #ひきこもり #死にたい (“#Not in Education, Employment, or Training (NEET) #Hikikomori #Want to die”)	24 (16.2)	
	Self-harm	Content that includes mentions or descriptions of self-harm and suicide	新品のカミソリ気持ちよすぎだろ⤴︎⤴ (“The brand-new razor blade feels so good⤴︎⤴”) 新品しか勝たん 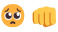 . (“New blades for the win 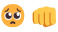 .”) #自傷行為 #不登校 #od (“#Self-harm #Not going to school #od”) #不登校と繋がりたい (“#I want to connect with school truants”) #リスカ #アムカ #レグカ (“#Wrist cutting #Arm cutting #Leg cutting”) #病み垢 #病み垢女子 (“#Account characterized by mental sickness #Girl with account characterized by mental sickness”) #病み垢女子さんと繋がりたい (“#Want to connect with girls with accounts characterized by mental sickness”) #病み垢さんと繋がりたい (“#Want to connect with people with accounts characterized by mental sickness”)	7 (4.7)	
	Developmental and learning disorders	Content that includes the mention of other developmental and learning disorders alongside “hikikomori” syndrome	#不登校 なのにも動じないで普通に接してくれるのはとても有難いんだけど #非同期発達 の事は、特に勉強面に関しては言い辛いから一寸困る。 こちらは正直に話しても良いんだけどホントに時々態度急変する人が居るから面倒臭くて試す気にはなれない。 “I’m very grateful that you don’t get upset and treat me normally even though I’m not going to school, but it’s a bit difficult to talk about #asynchronous development, especially when it comes to studying. I can be honest about this, but there are some people whose behavior changes suddenly after finding out, so I find it annoying and I don’t feel like risking it.”	3 (2)	
**Social determinants**					111 (75)
	School	Content that includes mentions of school and education, often through the act of missing school or unconventional alternatives to public school	娘がチャレンジの問題でつまずいて癇癪。 学校行ってる子達と比べたら圧倒的に解いてる問題数が違うからすぐにつまずく。 適室での勉強も家庭教師も拒否。 今自室で『自分だけの力でやってみせる』ってヤケになって勉強しに行った。もう限界でしょうよ 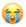 . どうすりゃいいの 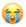 . (“My daughter stumbles over a challenging problem and has a tantrum. Compared to kids who go to school, the number of problems she solves is greatly less, so it’s easy to get stuck. She refuses to study in a proper room or have a private tutor. She just said, ‘I’ll do it on my own’ and went to study in her room. It’s probably at its limit 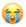 . What should I do 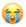 .”) #不登校 #不登校の親 (“#Not going to school #Parents of children not going to school”)	103 (69.6)	
	Work	Content that includes mentions of work, often through the act of missing or quitting work	親から「ウーバーイーツでも何でもやれ」「死に物狂いでやるしかないだろう」と言われた。(“My parents told me, ‘Find a job, work for Uber Eats if you need to’ and ‘Work as if your life depends on it.’”) 仕事するために”死に物狂い”になる必要がある状況って何だろう。(“In what situation would you need to be ‘desperate’ to do your job”) 仕事するために生きているわけでもないし。(“I don’t live to work.”) 親から何か言われるたび、死んだほうがマシだとしか思えない。(“Every time my parents say something to me, all I can think is that I would be better off dead.”) #ニート #ひきこもり #死にたい (“#NEET #hikikomori #Want to die”)	8 (5.4)	
**Awareness**					33 (22.3)
	Education	Content that includes active forms of providing education about “hikikomori” syndrome to the public or active advocacy	#不登校 #ひきこもり #ニート 今振り返れば。 今の自分なら。 そう言えるくらいに全部の経験が今に繋がる。 今どこかで悩んでいるおかあさんへ。 当事者さんへ。 あなたは大丈夫。 ひとりじゃない。 あなたはあなた。 他の誰かは他の誰か。 みんな違っていいんだよ。 “#Not going to school #Hikikomori #NEET Looking back now, I can say that all of my experiences have led me to where I am today. Dear mothers and other people who are worried right now. You are ok. You are not alone. You are you. Someone else is someone else. It’s okay for everyone to be different.”	3 (2)	

The social determinants parent domain was mentioned in 75% (111/148) of the tweets relevant to *hikikomori* experiences. Mentions of school or being “不登校” (“not going to school”) were common (103/111, 92.8%), reflecting the younger demographic of those posting about or experiencing *hikikomori* syndrome on Twitter. Although the age of users is difficult to determine, many individuals who sought out a human connection or social interaction on the platform requested relationships within an age range (eg, middle schoolers or high schoolers). Topics such as the lack of friendship due to their isolated lifestyle or their inability to go to school were discussed. Caregivers on Twitter discussing *hikikomori* syndrome were mainly parents of youth who were also “不登校” (“not going to school”). In addition, these caregivers displayed a sense of responsibility to improve their children’s lives or ease their difficulties and pain, observed through their active participation in seeking help. As a result, there were many secondhand experiences or caregiver community users detected in these tweets (71/111, 64%). Topics discussed included alternative education opportunities (eg, “フリースクール” [schools dedicated to children who fail to fit into conventional school systems in Japan] or “家庭教師” [“home or private tutoring”]), parenting philosophies, and specific experiences and advice regarding *hikikomori* syndrome. Less common were mentions of work and *hikikomori* (8/111, 7.2%). Tweets identified within this subtheme expressed an even greater disconnect from society and personal accounts of struggling to come to terms with lack of financial independence. Overall, within this parent domain, we found that *hikikomori* syndrome is heavily intertwined with the ideas of “ニート” (“not going to work”) and “不登校” (“not going to school”) in Japanese society. Even when tweet content alluded to a *hikikomori* lifestyle, many preferred the terms “ニート” and “不登校” (“not going to work” and “not going to school”) when self-identifying over explicitly identifying themselves as having *hikikomori* syndrome. The hashtags “#ニート” and “#不登校” were observed frequently with or even synonymously to *hikikomori* (“#引きこもり” or “＃ひきこもり”).

Within the awareness parent domain, individuals sought to reduce stigma regarding *hikikomori* syndrome by spreading awareness about the condition (33/148, 22.3%). Users spread awareness through Twitter primarily in 2 ways. Some tweets portrayed the syndrome positively by clearing misconceptions that previously created apprehensiveness toward *hikikomori* syndrome or by drawing attention to the benefits of the lifestyle (eg, having less disputes between family members and, consequently, having a more peaceful and connected family life in certain circumstances). Other tweets highlighted the negative attitudes toward the syndrome and aimed to reduce stigma by portraying stigmatization of *hikikomori* in a negative context (eg, explaining how a friend’s negative comments were morally unacceptable). This was frequently observed through users recalling an experience in which an individual experiencing *hikikomori* syndrome or their caregivers faced shame for their condition. Tweets were not always targeted toward the public and, instead, aimed to reduce internal shame of the syndrome by addressing users with similar experiences. Tweets within the education subtheme (3/33, 9%) were characterized by users providing knowledge to the public through digital flyers, meetings, or other active forms of creating awareness, directly addressing their audience in the process. Tweets within this subtheme were only posted by caregivers and in secondhand accounts as explicit advocacy and education often requires contact with the public. Although it was uncommon, some caregivers used the platform to educate and act in the role of mediator between the isolated population and the uninformed public.

In general, those experiencing *hikikomori* and their caregivers used Twitter to either share experiences and opinions with the public through 1-way communication (personal anecdotes, emotional ranting, and advocacy) or increase social interaction and discussion through 2-way communication (seeking social support and seeking and giving advice). Through 1-way communication, those experiencing *hikikomori* disclosed important and often personal information on their lifestyle or used the platform as a means to discuss and cope with their struggles. Caregivers often shared their own experiences with family members with *hikikomori* syndrome and also worked to directly reduce stigma. Through 2-way communication, those experiencing *hikikomori* found like-minded individuals on the web to connect with. Caregivers also exchanged advice and information to better support individuals experiencing *hikikomori*. We found more 1-way communication (53/71, 75% of firsthand accounts and 54/77, 70% of secondhand accounts) than 2-way communication in the tweets analyzed. However, the audience and motive of the tweets were often unspecified.

## Discussion

### Principal Findings

This study collected and analyzed 2,018,822 tweets with terms related to *hikikomori* syndrome, a form of severe social withdrawal prevalent in Japan, and after conducting data filtering for more recent posts posted between January 2021 and January 2023 and topic modeling for detection of prevalent themes, we found both first- and secondhand experiences reported among Japanese-language tweets (148/2,018,822, 0.01%) from 89 unique users. Among our sample, we found that 48% (71/148) of tweets discussing their experiences with *hikikomori* syndrome were firsthand accounts of the challenges associated with their daily lives, whereas 52% (77/148) were identified as secondhand accounts mainly from caregivers. Within both first- and secondhand reports, the parent categories identified were clinical symptoms, social determinants, and awareness.

Within the 3 parent domains, we found 8 subthemes, which included users describing firsthand and secondhand experiences with *hikikomori* symptoms, including anxiety, depression, social isolation, self-harm, and developmental disorders, as well as discussion related to missing school or work, a commonly reported manifestation of *hikikomori* [1]. Twitter users in this study also shared advocacy and educational awareness related to the syndrome and sought out connections with other web-based users. Similarly to previous research, this study found a variety of topics. Common use of personal anecdotes and other detected topics such as social support, exchange of advice, and stigma are in line with and further support existing research findings, emphasizing the potential value of social listening–related *hikikomori* discourse on social media platforms where *hikikomori* communities interact [22]. Our findings provide additional novel context by focusing on first- and secondhand experiences of the syndrome to better characterize lived experiences with *hikikomori*. Previous studies have identified topics such as marketing, employment and educational opportunities, and medical and science topics related to the syndrome, which were excluded from this study [22,23].

This study provides additional context to the *hikikomori* literature and provides the first social media–based study to characterize web-based discussions from both the first- and secondhand perspectives in the Japanese language, specifically following the COVID-19 pandemic. Of the social media studies that have characterized lived experiences with *hikikomori*, some have focused on *hikikomori* in Western societies, including European countries, in which individuals who directly experience *hikikomori* were the most active users, in contrast to this study, in which secondhand posts were the most commonly detected overall (eg, caregivers or friends of those with *hikikomori* syndrome) [32]. While COVID-19 restrictions resulted in mandated social isolation to different degrees for people worldwide, there may have been more visibility of *hikikomori* symptoms by caregivers that may not have been otherwise observed before many of the public health restrictions during the pandemic. In turn, caregivers may have turned to social media to connect with others, seek advice about *hikikomori*, or spread awareness of the syndrome.

While our study found more secondhand experiences with *hikikomori* overall, within our clinical symptoms parent topic, we found an overwhelming majority of firsthand reporting of *hikikomori* (52/58, 90%). These findings may indicate that, when an individual is struggling with *hikikomori*, they are more likely to self-report their struggles with the syndrome and its associated symptoms on the web. Concerningly, as detected in this study, individuals may take to web-based platforms to report more severe symptoms and mental health struggles, such as suicidal ideation [33]. However, social media has increasingly represented a valuable way to detect depression and suicidal ideation and can provide rapid data for policy-level decisions, especially given the rise of mental health conditions during and after the pandemic [33]. As such, this finding may also represent shifting ideas and definitions regarding *hikikomori*, especially after the COVID-19 pandemic, a period characterized by social isolation, remote education, and increasing mental health concerns [26]. Furthermore, firsthand users detected in this study may take to web-based platforms as a way to discuss their own experiences and self-report *hikikomori*-related symptoms but appear to engage in less education, advocacy, or awareness raising compared to those with secondhand experiences based on our observations.

Platforms such as Twitter may be an advantageous and comfortable way for those with *hikikomori* syndrome to interact with others while in a lifestyle that lacks social interaction, especially during the mandated social distancing measures that aligned with the study period. Simultaneously, the results provide updated insights into the lives of those with *hikikomori* syndrome and others who support them, as well as into direct advocacy by those who are affected. The findings indicate that access to information on this syndrome through social media platforms can increase access to other individuals and broader online communities experiencing the syndrome, possibly facilitated by semianonymous and web-based conversations, which may otherwise be inhibited by physical barriers due to the isolating nature of *hikikomori*. By leveraging platforms such as Twitter, greater interactions within the community can potentially reduce internal stigma and shame, whereas greater interactions with the public can reduce external stigma toward the syndrome as a whole [34]. In addition, open discussion about experiences and resources available, both within the community and through interactions with the public, could lead to greater accessibility to those resources and more awareness and acceptance.

### Limitations

This study has certain limitations. First, it only evaluated data from publicly available content on Twitter and limited the analysis to Japanese-language tweets and tweets that were in both Japanese and English, which is not representative of general social media *hikikomori*-related discourse, including that occurring on other platforms such as Facebook, Reddit, TikTok, and Instagram. Hence, this study may fail to capture posts from individuals who have additional privacy settings or engage in conversations via private or direct messages due to the stigmatization of mental health issues. Furthermore, this study only analyzed tweets posted by users and not comments or other interactions between Twitter users in response to a tweet, which could have yielded additional discussion related to *hikikomori*. In addition, our period of data collection and analysis coincided with the COVID-19 pandemic, which significantly impacted individuals’ way of life and required social isolation. Hence, the volume and nature of *hikikomori* discussions on the web may have also been driven by the COVID-19 restrictions during the study period. This study likely underreported the total amount of *hikikomori*-related content within the dataset as we only coded tweets that were the most highly engaged with within selected topic clusters. This approach streamlines manual coding and allows for more efficient detection of relevant conversations but may exclude tweets that have low engagement. Furthermore, this study may have oversampled what is considered clinical *hikikomori* discussions due to variation in the colloquial meaning of *hikikomori*, the potential expansion of *hikikomori* to refer to less severe symptoms, and the reliance on self-reported accounts from web-based users and their perception of their or someone else’s experience with *hikikomori*. Hence, it is crucial to acknowledge that this study’s findings are specific to a subset of *hikikomori* accounts and content—those who consider themselves as experiencing *hikikomori* first- or secondhand. As such, it may not capture the diversity of *hikikomori* behaviors and attitudes and lacks generalizability to the overall population of those who experience it. In addition, although Twitter offers users a significant degree of anonymity through features such as customizable usernames and the option to create throwaway accounts for sensitive discussions, self-reported measures remain susceptible to recall bias and social desirability bias, which could lead to over- or underreporting of behaviors. Thus, tweets coded as *hikikomori* may in some instances be less representative of the clinical condition and more associated with the casual use of the term to describe non-*hikikomori* symptoms or may more broadly reflect the collective understanding of *hikikomori* as a concept in Japanese culture by those who do not actually have the condition from a clinical context. Future studies should explore multi-platform analysis for *hikikomori*-related discussions, combine social media data with other data sources (eg, focus groups and surveys), and use other data science approaches (eg, supervised machine learning and large language models) to better characterize *hikikomori* changes over a longer period both before and after the pandemic.

### Conclusions

Understanding culturally specific self-reported symptomology through social media studies may offer insights into the convergence and divergence of cross-national *hikikomori* experiences. In addition, commonalities in experiences and rhetoric provide insights into the Japanese public’s view of *hikikomori* and its prevalence in Japanese society. The findings of this study also have potential clinical implications. As *hikikomori* is increasingly recognized as a global concern, clinicians may look to web-based platforms and discussion forums to understand modern manifestations of the syndrome and the collective understanding of the concept in different cultural contexts (both in Japan and other cultures experiencing *hikikomori*), especially as a standardized definition and criteria are evolving [4]. This study may also provide additional evidence that online support groups may be well received among those with *hikikomori* and could provide clues on how to help relieve adverse experiences associated with social withdrawal as well as provide social support for those caring for someone with *hikikomori* [32]. These results may also justify the need to increase telehealth consultations in the post–COVID-19 era regarding *hikikomori* screening and possible diagnosis. However, increasing participation in digital care and support opportunities for patients with *hikikomori* syndrome should be afforded careful consideration to ensure that that same technology does not facilitate further social isolation if not used correctly or in a culturally appropriate manner [35,36].

## Appendix

Multimedia Appendix 1 Study keyword selection and rationale for use of the keywords.

## Abbreviations

API: application programming interface
BERT: Bidirectional Encoder Representations From Transformers
JMHLW: Japanese Ministry of Health, Labor, and Welfare

## Data Availability

The datasets generated or analyzed during this study are available from the corresponding author upon reasonable request.
