# Supplementary material for: Characterizing Experiences With Hikikomori Syndrome on Twitter Among Japanese-Language Users: Qualitative Infodemiology Content Analysis
Source: JMIR Infodemiology. 2025 Feb 24;5:e65610. doi: 10.2196/65610 (PMC11894343; doi:10.2196/65610)
Supplement: Multimedia Appendix 1 [file infodemiology_v5i1e65610_app1.docx]

**Supplementary File**

**Supplement Table 1. Study keyword selection and rationale for use.**

| Keyword | Translation | Rationale for Study Use |
| --- | --- | --- |
| #引きこもり | Hikikomori | As the kanji (漢字)version of “hikikomori”, it opens up the study to different variations of the same word. |
| #ひきこもり | Hikikomori | As the hiragana (ひらがな)version of “hikikomori”, it opens up the study to different variations of the same word. |
| #hikikomori | Hikikomori | As the English and the romaji （ローマ字）version, it opens up the study to different variations of the same word. |
| #ニート | Unable to go to work | The concept is very interlinked and often synonymous to hikikomori syndrome in Japanese society. It may be preferred as self-identification over “hikikomori” itself. |
| #脱ひきこもり | Leaving hikikomori lifestyle behind | Individuals who are experiencing hikikomori syndrome but are seeking to lead a non-hikikomori lifestyle may have different uses of the platform and could add to a more wholistic understanding of the syndrome. |
| #不登校 | Unable to go to school | The concept is very interlinked and often synonymous to hikikomori syndrome in Japanese society. It may be preferred as self-identification over “hikikomori” itself. |
| #自宅警備員 | Home guard | As a more casual term, it can be used in first hand accounts, sometimes preferred over more stigmatized terms such as “hikikomori”. Thus, it opens up the study to a wider range of first hand and second hand accounts. |
